# Perspective and Costing in Cost-Effectiveness Analysis, 1974–2018

**DOI:** 10.1007/s40273-020-00942-2

**Published:** 2020-10-20

**Authors:** David D. Kim, Madison C. Silver, Natalia Kunst, Joshua T. Cohen, Daniel A. Ollendorf, Peter J. Neumann

**Affiliations:** 1 Center for the Evaluation of Value and Risk in Health, Institute for Clinical Research and Health Policy Studies, Tufts Medical Center, 800 Washington St, Box 063, Boston, MA 02111 USA; 2 Department of Medicine, Tufts University School of Medicine, Boston, MA USA; 3 Department of Health Management and Health Economics, University of Oslo, Oslo, Norway; 4 Cancer Outcomes, Public Policy and Effectiveness Research (COPPER) Center, Yale University School of Medicine, New Haven, CT USA; 5 LINK Medical Research, Oslo, Norway

## Abstract

**Objective:**

Our objective was to examine perspective and costing approaches used in cost-effectiveness analyses (CEAs) and the distribution of reported incremental cost-effectiveness ratios (ICERs).

**Methods:**

We analyzed the Tufts Medical Center’s CEA and Global Health CEA registries, containing 6907 cost-per-quality-adjusted-life-year (QALY) and 698 cost-per-disability-adjusted-life-year (DALY) studies published through 2018. We examined how often published CEAs included non-health consequences and their impact on ICERs. We also reviewed 45 country-specific guidelines to examine recommended analytic perspectives.

**Results:**

Study authors often mis-specified or did not clearly state the perspective used. After re-classification by registry reviewers, a healthcare sector or payer perspective was most prevalent (74%). CEAs rarely included unrelated medical costs and impacts on non-healthcare sectors. The most common non-health consequence included was productivity loss in the cost-per-QALY studies (12%) and patient transportation in the cost-per-DALY studies (21%). Of 19,946 cost-per-QALY ratios, the median ICER was $US26,000/QALY (interquartile range [IQR] 2900–110,000), and 18% were cost saving and QALY increasing. Of 5572 cost-per-DALY ratios, the median ICER was $US430/DALY (IQR 67–3400), and 8% were cost saving and DALY averting. Based on 16 cost-per-QALY studies (2017–2018) reporting 68 ICERs from both the healthcare sector and societal perspectives, the median ICER from a societal perspective ($US22,710/QALY [IQR 11,991–49,603]) was more favorable than from a healthcare sector perspective ($US30,402/QALY [IQR 10,486–77,179]). Most governmental guidelines (67%) recommended either a healthcare sector or a payer perspective.

**Conclusion:**

Researchers should justify and be transparent about their choice of perspective and costing approaches. The use of the impact inventory and reporting of disaggregate outcomes can reduce inconsistencies and confusion.

**Electronic supplementary material:**

The online version of this article (10.1007/s40273-020-00942-2) contains supplementary material, which is available to authorized users.

## Key Points for Decision Makers

**Table:** 

The analytic perspective assumed in cost-effectiveness analysis determines which costs and benefits are included. Despite its importance, study authors often mis-specified or did not clearly state the perspective used.
When a societal perspective was used, authors often did not apply it as broadly as intended. Only a few non-health consequences, such as productivity or transportation, were considered, whereas broader non-healthcare sector impacts were seldom examined.
The use of a healthcare payer or a healthcare sector perspective persists in most published studies and national guidelines because “relevant” non-health benefits and costs are often difficult to define and may depend on the context. The consistent use of the impact inventory and reporting of disaggregate outcomes can help reduce discrepancies across analyses capturing non-health consequences.

## Background

Practice guidelines for cost-effectiveness analysis (CEAs) emphasize the importance of the analytic perspective assumed in the analysis because it determines which costs and benefits are included [1–4]. The perspective may reflect a patient, a specific payer (public or private), the entire healthcare sector, or all of society. The choice of perspective and included cost components may have a substantial impact on the cost-effectiveness of interventions and, consequently, policy and resource allocation decision making [5–7].

Ideally, the relevant audience—i.e., the population “on whose behalf are decisions made”—influences the choice of perspective [8]. Practice guidelines that endorse a broader societal perspective argue that considering everyone affected and counting all benefits and costs, regardless of who gains or loses, can provide the basis for fair decisions in the public interest [1, 8, 9]. A societal perspective means that all resources (both health related and not) associated with the intervention should be identified, measured, and valued. Some guidelines, such as the Original Panel on Cost-Effectiveness in Health and Medicine in 1996 [8], have suggested the use of a societal perspective as a reference case analysis to encourage comparability and consistency of the analytic perspective across CEAs.

However, since publication of the Original Panel’s guideline, awareness of challenges in how to implement a “societal” perspective has grown. Recognizing practical difficulties and the revealed preferences of some policy makers to support narrower perspectives, the Second Panel on Cost-Effectiveness in Health and Medicine revised the original guidelines in 2016. A major change in the Second Panel’s guideline includes two reference case analyses, from both a healthcare sector and a societal perspective [10]. Along with the recommendation, the Second Panel advises the use of an “impact inventory” to help standardize practice and reduce confusion. The impact inventory is a structured table listing an intervention’s health and non-health consequences, including those falling outside the formal healthcare sector. Although the Second Panel’s updated recommendation for the inclusion of an impact inventory was published in 2016, to our knowledge, its impact on practice has not yet been evaluated.

Understanding past and current practices can shed light on the gap between guideline recommendations and actual behavior and provide future directions for improvement. Although a few studies have documented variations in perspective used and costing approaches [11–13], the number of published CEAs has increased substantially in recent years, and many countries have formally adopted a health technology assessment (HTA) process [14]. We sought to examine the use of perspective and costing approaches, including the use of multiple perspectives and the impact inventory, in published CEAs through 2018 and their impact on reported incremental cost-effectiveness ratios (ICERs). In addition, we reviewed 45 country-specific guidelines to examine recommended analytic perspectives in HTA.

## Methods

### Working Definitions

In this paper, we avoid the widespread notions of “direct” and “indirect” costs as it is often difficult to differentiate between direct and indirect consequences of disease and its treatment. Also, a range of possible consequences could be deemed “direct” depending on the perspective of the analysis (e.g., all non-health consequences that influence public welfare can be considered “direct” from a societal perspective) [15]. Instead, we use the term “non-health consequence” to capture both medical and non-medical resources consumed. The non-health consequences include patient time, transportation costs, caregiver time, productivity, and other non-healthcare sector impacts on education, criminal justice, housing, and environment. Based on this categorization, we applied the following definition of analytic perspectives:

*Healthcare payer*: This perspective includes only those monetary costs (e.g., treatment costs and other health service resource use associated with disease management) incurred by a (typically third party) healthcare payer (e.g., Medicare/Medicaid, British national health service, a health maintenance organization, etc.).

*Healthcare sector*: This perspective is similar to the healthcare payer perspective but accounts for all monetary costs of healthcare, regardless of who bears the cost. A key distinction between the healthcare sector and healthcare payer perspectives is that the healthcare sector perspective includes patients’ out-of-pocket costs.

*Limited societal*: This perspective accounts for cost components beyond those captured by the healthcare sector perspective, including patient time, patient transportation, unpaid caregiver time, and productivity loss. It excludes spillover impacts affecting sectors other than healthcare, such as education.

*Societal*: A societal perspective is broader than the limited societal perspective. It represents the overall public interest by including all resources that could be used for other purposes. The analysis accounts for cost impacts affecting at least one of these other sectors, such as the environment, education, or the justice system.

*Not stated/could not be determined*: This designation indicates that the authors did not provide sufficient information for us to determine the type of costs or benefits included in the analysis.

### Data

We analyzed data from the Tufts Medical Center’s CEA Registry (a database of cost-per-quality-adjusted-life-year [QALY] studies; available at www.cearegistry.org) and the Global Health CEA (GHCEA) registry (an open-access database of cost-per-disability-adjusted-life-year [DALY] studies; available at www.ghcearegistry.org). Both registries contain information from English-language, PubMed-indexed original CEAs (i.e., excluding reviews, editorials, or methodological articles) from 1974 through 2018, which report at least one cost-per-QALY gained or cost-per-DALY averted ratio, including interventions with cost-saving (i.e., dominant) or dominated ratios. We identified 6907 cost-per-QALY and 698 cost-per-DALY studies, reporting 19,946 and 5572 intervention-specific ICERs, respectively. All of the ratios available in the registries were converted to $US, year 2018 values based on currency conversions [16] and inflation adjustments using the consumer price index [17].

Both registries applied structured and standardized data collection processes to ensure the consistency and quality of the data. Methodological details are available elsewhere [18, 19], including the registry website, but we briefly summarized the process. After an initial literature search, at least two registry reviewers independently conduct abstract screening to determine eligibility for the full review. Among articles selected for the full review, each reviewer used a prespecified data collection form to extract information on a wide range of variables, including author affiliation, study country, study sponsorship, intervention, comparator(s), target population, perspective, time horizon, discounting rate, cost-effectiveness ratios, etc.[20]. Throughout the process, the reviewers engaged in a consensus discussion to address any discrepancies before moving onto the next step.

### Main Analyses

We first investigated the use of perspective and its changes over time among cost-per-QALY and cost-per-DALY studies, including any discrepancies between the perspective reported by the study authors and the perspective used in the analysis, as determined by our registry reviewers. Registry reviewers extracted information pertaining to the analytic perspective stated by authors, and two trained reviewers reclassified the perspective based on their assessment of the presented analysis and cost components included. For example, if an author stated that the analysis used the societal perspective but did not describe the inclusion in the analysis of any spillover costs affecting other sectors (e.g., education, housing, judicial), then the reviewer designated the actual perspective as limited societal. For comparative analyses, we excluded papers for which the perspective was not stated/could not be determined and aggregated the remaining papers into either societal/limited societal or healthcare sector/payer.

We also examined how often published CEAs included cost components beyond those traditionally included in an analysis from a healthcare sector perspective. Due to changes in the data collection methodology for the CEA registry [20], we were only able to obtain information on specific cost components included in cost-per-QALY articles published since 2013 (*N* = 2839 of 6907). On the other hand, we retained that information for all 698 cost-per-DALY studies in the GHCEA registry.

We compared cost components included in cost-per-QALY versus cost-per-DALY studies and then investigated how the choice of the analytic perspective influences the reported ICERs across different intervention types and study sponsors. To further examine the association between the inclusion of cost components and reported ICER values, we analyzed a subset of cost-per-QALY studies (those published in 2017–2018) and cost-per-DALY studies that reported cost-effectiveness ratios from both a healthcare sector and a societal perspective. Using ratios drawn from the same study makes it possible to isolate the impact of altering the perspective while holding all other aspects of the CEA unchanged. Additionally, we examined the inclusion of the impact inventory, which is recommended by the Second Panel.

### Secondary Analysis of the National Health Technology Assessment (HTA) Guidelines

We also reviewed 45 national, country-specific guidelines governing economic evaluation or HTA to examine differences in recommended analytic perspectives across countries. We identified the national guidelines from a literature search, online databases, and published reviews of HTA guidelines [21–27]. We classified the recommended perspectives based on the cost components recommended for inclusion and from the guideline perspective statements. In some cases, although the guidelines recommended a healthcare sector perspective, they only accounted for healthcare payer costs, and we reclassified them as describing a healthcare payer perspective. Additionally, among the top five countries with most CEAs produced (the USA, the UK, Canada, Netherlands, and Australia), we examined differences in the proportions using the societal perspective and its consistency with the national guidelines.

## Results

### Main Analysis 1: Perspective Used and Costing Approaches

**Fig. 1:**
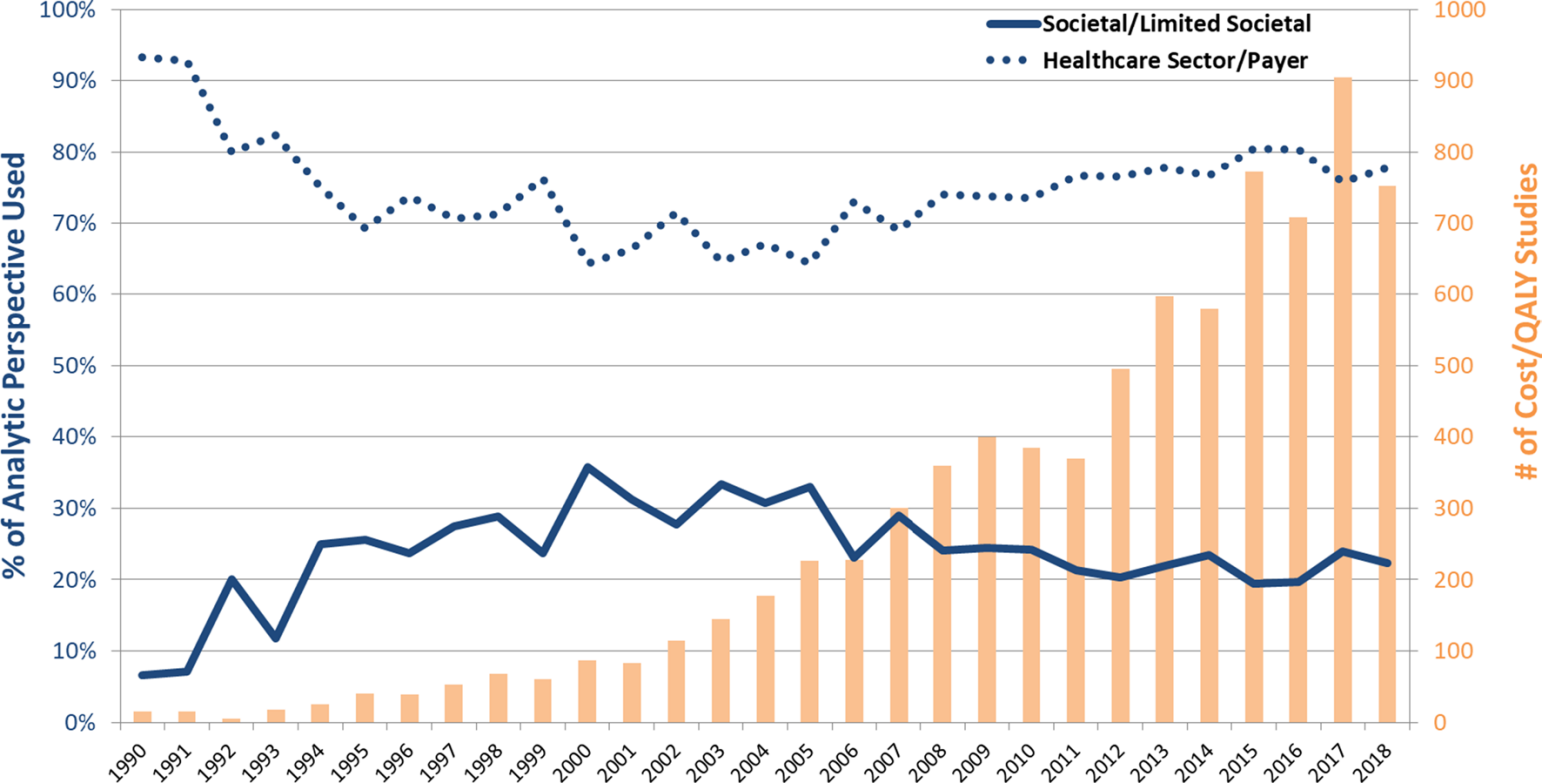
Trends in analytic perspectives used in cost-per-QALY studies: 1974–2018 (*N* = 6,907). With relatively small number of cost-per-QALY studies published prior to 1990 (*n* = 18, 0.3%), the Figure shows the data points since 1990. A similar figure for cost-per-DALY studies is available in the Online Supplement Figure A

Study authors often mis-specified the perspective used, and 20% of CEAs (both cost-per-QALY and cost-per-DALY studies) did not clearly state the study perspective. After the registry reviewers reclassified the study perspective based on types of costs or benefits evaluated, the study perspective was unable to be determined in only 7% of CEAs. Based on the perspective as determined by registry reviewers, published CEAs most often used a healthcare sector perspective (75% of cost-per-QALY studies and 65% of cost-per-DALY studies). Our data indicate that cost-per-QALY study authors reported a societal/limited societal perspective more frequently than it was actually used, as determined by registry reviewers. In contrast, cost-per-DALY study authors tended to underreport the use of a societal/limited societal perspective by mis-specifying it as a healthcare sector perspective (Table 1). While the use of the healthcare perspective in cost-per-QALY studies has increased over the past decade (Fig. 1), the perspective used in cost-per-DALY studies has not changed significantly over time (Electronic Supplementary Material [ESM] A: Table A and Fig. A).

**Table 1:** Perspective used in published cost-effectiveness analyses, 1974–2018 (*N* = 6907)

Perspectives	Cost per QALY gained (1974–2018)		Cost per DALY averted (1995–2018)		Both types of studies (1974–2018)	
	As stated by authors	As determined by reviewers	As stated by authors	As determined by reviewers	As stated by authors	As determined by reviewers
Societal/limited societal	1556 (22.5)	1165 (16.9)	198 (28.4)	222 (31.9)	1754 (23.1)	1387 (18.2)
Healthcare sector/payer	3846 (55.7)	5160 (74.7)	343 (49.1)	457 (65.4)	4189 (55.1)	5617 (73.9)
Not stated/could not be determined^a^	1408 (20.4)	527 (7.6)	142 (20.4)	10 (1.4)	1550 (20.4)	537 (7.1)
Other	97 (1.4)	55 (0.8)	15 (2.2)	9 (1.3)	112 (1.5)	64 (0.8)
Total	6907 (100)	6907 (100)	698 (100)	698 (100)	7605 (100)	7605 (100)

Data are presented as *N* (%)

*DALY* disability-adjusted life-year, *QALY* quality-adjusted life-year

^a^Authors did not provide sufficient information to determine types of costs or benefits evaluated

**Fig. 2:**
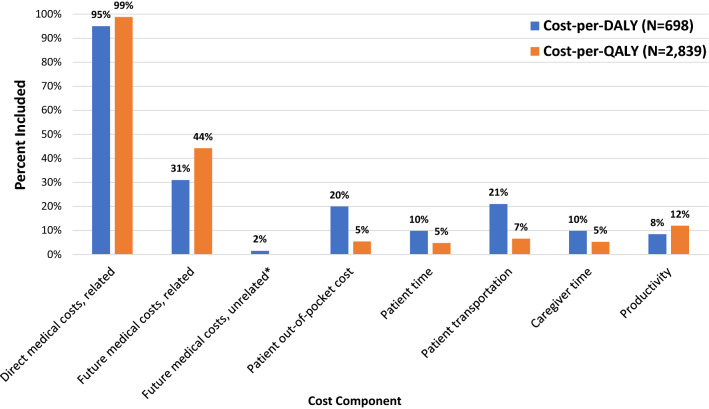
Cost components included in published cost-effectiveness analyses. *Future unrelated medical cost data was not available for cost-per-QALY studies. Due to the changes in the data collection methodology, we were only able to obtain cost components included in cost-per-QALY literature published since 2013 (*N* = 2,839 out of 6,907)

Although most studies included immediate medical costs associated with the intervention, less than half included future medical costs related to the intervention (Fig. 2). While future unrelated medical cost data were not available in the cost-per-QALY registry, cost-per-DALY studies rarely included future unrelated medical costs (i.e., background medical costs of treating conditions in added years of life). Cost-per-DALY studies were more likely to include at least one cost component outside of the healthcare sector (36 vs. 10% cost-per-QALY studies). The most commonly included non-health consequence was productivity (12%) in cost-per-QALY studies and patient transportation (21%) in cost-per-DALY studies. Published CEAs rarely examined impacts affecting other non-healthcare sectors, such as criminal justice, education, and housing (2% among cost-per-QALY studies, and 5% among cost-per-DALY studies).

### Main Analysis 2: Association Between Study Characteristics and Incremental Cost-Effectiveness Ratio

Of 19,946 cost-per-QALY ratios in our sample, the median ICER was $US26,000/QALY (interquartile range [IQR] 2900–110,000), and 18% of ratios were cost saving and QALY increasing. Of 5572 cost-per-DALY ratios, the median ICER was $US430/DALY (IQR 67–3400), and 8% of ratios were cost saving and DALY averting. Pharmaceuticals were the most common intervention type examined in both types of studies, followed by immunizations (29.7%) and health education (26.3%) among cost-per-DALY studies, and medical or surgical procedures (19.4%) and care delivery (8.2%) among cost-per-QALY studies.

Overall, among cost-per-QALY ratios calculated from a healthcare sector perspective (76%), the median ICER was $US25,000/QALY (IQR 3100–100,000); the corresponding median ICER among the ratios calculated from a societal perspective was $US30,000/QALY (IQR 2300–150,000). Among cost-per-DALY ratios calculated from a healthcare sector perspective (74%), the median was $US460/DALY (IQR 74–3500); it was $US345/DALY (IQR 51–2700) in societal perspective analyses (ESM A: Table B and Fig. B).

In the cost-per-QALY literature, drug and device industry-sponsored studies consistently reported lower median ICERs than non-industry-sponsored studies from both perspectives (i.e., $US18,000 vs. 31,000/QALY from a healthcare sector perspective and $US27,000 vs. 36,000/QALY from a societal perspective). We found a paucity of cost-per-DALY ratios from industry-sponsored studies (4%, *n* = 197), and none of the industry-sponsored cost-per-DALY ratios reported that the interventions were “dominated” (i.e., less effective and more costly than its comparator; ESM A: Tables C and D).

### Main Analysis 3: The Use of Impact Inventory and Multiple Perspectives

For the years 2017–2018, we identified 11 cost-per-QALY studies (1.0%) that included the impact inventory table (we did not collect this information among cost-per-DALY studies). Nine of 11 studies came from the USA (*n* = 5) or the UK (*n* = 4), from which at least one member of the Second Panel came. Two other studies were from Ireland and Switzerland.

We found that 16 cost-per-QALY studies (1.4%) reported a total of 68 cost-per-QALY ratios from a healthcare sector and a societal perspective (34 ratios from each perspective), and five cost-per-DALY studies (0.7%) reported a total of 72 cost-per-DALY ratios (36 ratios from each perspective). Seven of 16 cost-per-QALY studies reported one ratio from each perspective [28–34], whereas two studies reported five and six intervention-specific ratios from each perspective, respectively [35, 36]. Four of five cost-per-DALY studies reported one or two ratios from each perspective [37–40], and one multicountry study reported 31 ratios from each perspective [41].

**Fig. 3:**
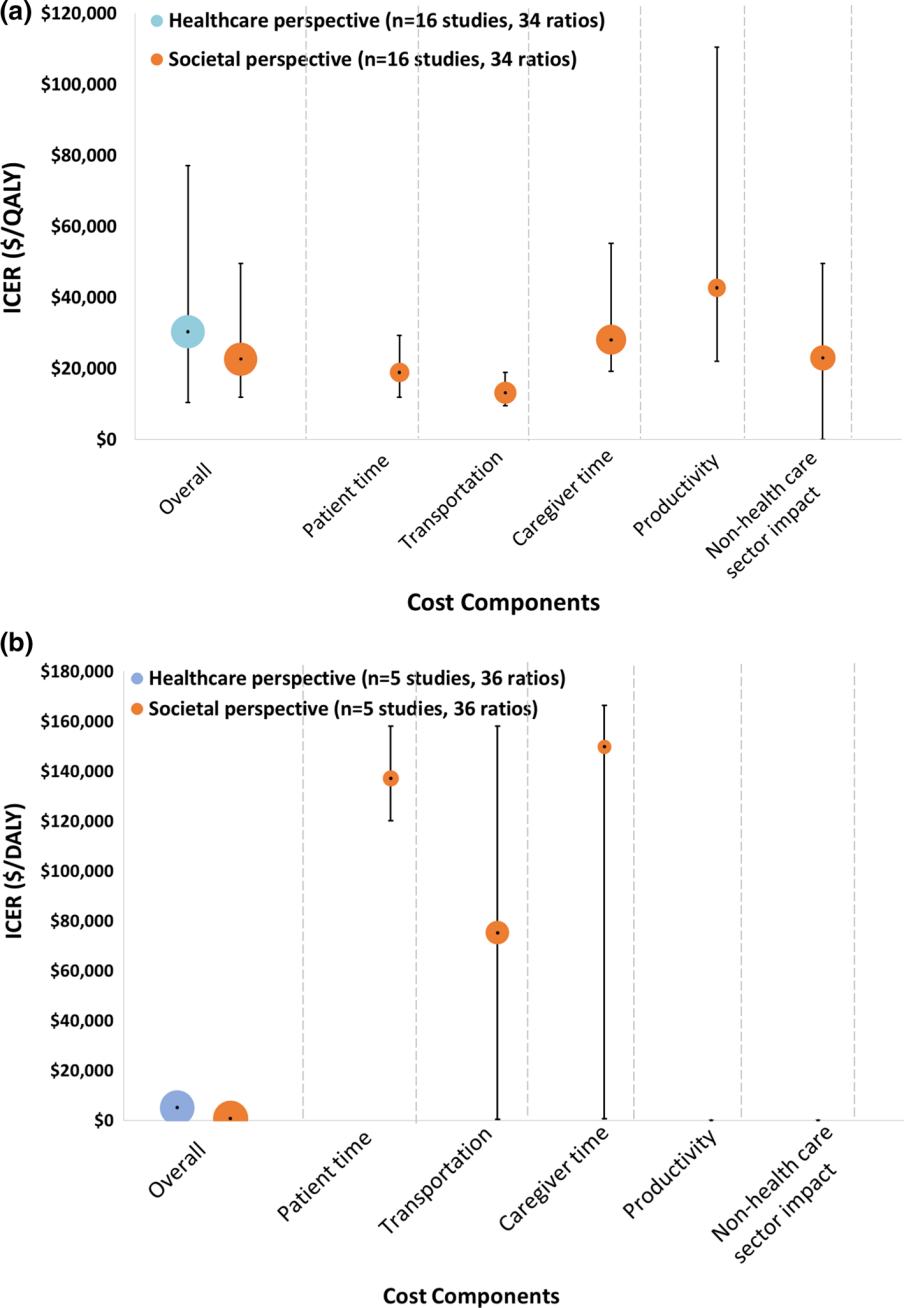
Incremental cost-effectiveness ratios by the inclusion of non-health components. Note: The size of the circle represents the volume of included studies for each perspective non-health components. For studies that included each cost component, the center of the circle denotes the median incremental cost-effectiveness ratios (ICER) while the lines extend to the 25th and 75th percentiles, the inter-quartile range (IQR). Due to the wide IQR of some ICERs, Lines extending to the x-axis represent interventions that were cost-saving at the 25th percentile. None of the cost-per-DALY studies included productivity or non-healthcare sector costs

The studies that reported ratios from both perspectives had lower (more favorable) median ICERs from a societal perspective ($US22,710/QALY [IQR 11,991–49,603] and $US804/DALY [IQR {cost saving}–3277) than from a healthcare sector perspective ($US30,402/QALY [IQR 10,486–77,179] and $US5119/DALY [IQR 3186–5607]). Due to expensive treatments relative to the non-health benefits, three studies that included specific non-health consequences, such as productivity or caregiver cost, reported higher median ICERs than those from 16 studies that used a healthcare sector perspective (Fig. 3). However, within these studies [31, 42, 43], the median ICER from a societal perspective was substantially lower than that from a healthcare perspective ($US42,745 vs. 123,514/QALY, respectively).

### Secondary Analysis: Recommended Perspectives by National Guidelines on HTA

**Fig. 4:**
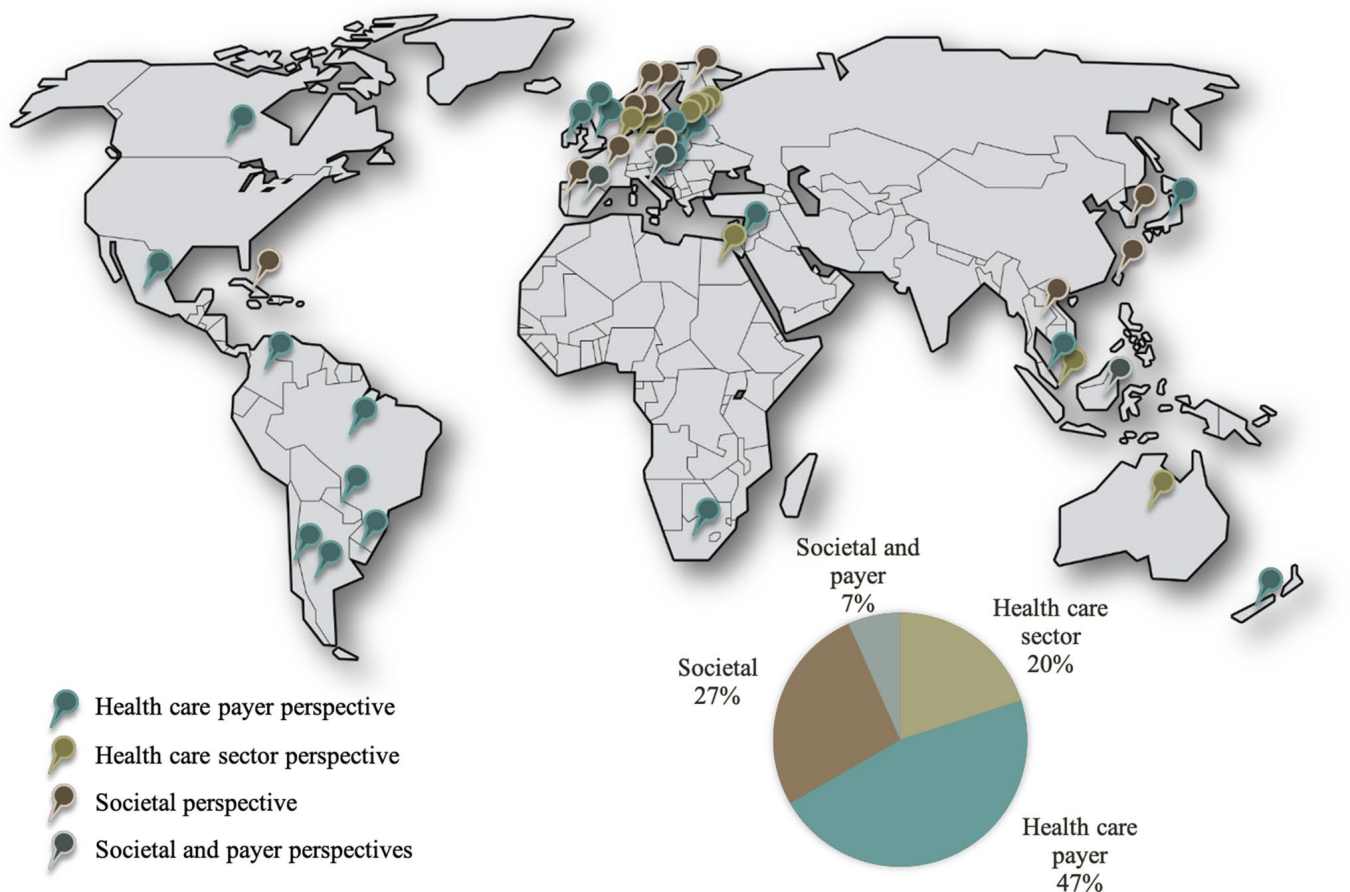
Recommended perspectives across 45 national guidelines on health technology assessment. Note: The Figure used the world map frame available at https://pngimg.com/imgs/miscellaneous/world_map/

Most government guidelines (67%) recommend either a healthcare payer or healthcare sector perspective as the reference perspective (Fig. 4 and ESM A, Table E). Eighteen countries (40%) endorse the healthcare payer perspective with supplementary use of a broader societal or another perspective, and three additional countries (i.e., Israel, New Zealand, and Scotland; 7%) recommend using *only* a healthcare payer perspective. A societal perspective, with or without a supplementary perspective, is the primary analytic perspective in 12 countries (27%). Three of the reviewed countries (Indonesia, Italy, and Spain; 7%) recommend consideration of both societal and healthcare payer perspectives. For the subanalysis of the most recently published or updated guidelines in the past 5 years, the proportion of guidelines recommending a healthcare payer or healthcare sector perspective was higher than the overall rate of 67% (30 of 45 guidelines published in 1998–2019) at 71% (15 of 21 guidelines published in 2015–2019; ESM A: Table E, and ESM B).

Our country-specific analysis showed that the proportion of cost-per-QALY studies for the Netherlands from a societal perspective was 49% (232 of 476), almost three times higher than the average among the entire cost-per-QALY literature (16.9%). Among the top five CEA-producing countries, the Netherlands was the only one that recommended a societal perspective, whereas the UK, Canada, and Australia recommended a narrower (national healthcare payer or healthcare sector) perspective. Only 14% (99 of 519) and 10% (118 of 1225) of Australia- and UK-based cost-per-QALY studies were conducted from a societal perspective, respectively (ESM A: Fig. C).

## Discussion

Using an updated database of published CEA, we found that key study attributes—including perspective and cost components considered—varied substantially across types of evaluation and setting. In particular, we found a considerable discrepancy between the author’s stated perspective and our reviewers’ judgments about the perspective used. It appears that some researchers are unclear on what cost components should be included in a CEA conducted from a societal perspective. When analysts did use a societal perspective, they often did not apply it as broadly as the Second Panel and other experts intended. Only a few non-health consequences related to the intervention—for example, productivity or transportation—were considered, whereas broader non-healthcare sector impacts—for example, those affecting the criminal justice system or education—were seldom examined.

We also found substantial variation among reported cost-effectiveness ratios by study characteristics and costing approaches. Specifically, the analysis of the subset of cost-per-QALY and cost-per-DALY studies that included multiple perspectives found that the inclusion of broader non-health consequences generally resulted in lower (more favorable) cost-effectiveness ratios. For example, pharmacotherapy for patients with alcohol use disorder becomes more cost-effective when improved outcomes that go beyond the healthcare sector, such as improved productivity or reduced alcohol-related motor vehicle accidents, are accounted for [44]. However, this may not always be the case as the inclusion of non-health consequences can sometimes result in less favorable cost-effectiveness ratios (e.g., the early discharge of patients from hospitals can lead to higher informal caregiver costs and thus less favorable cost effectiveness).

However, the use of a healthcare payer or a healthcare sector perspective persists in most published studies and national guidelines. By taking a narrower perspective, some aspects of societal value elements can be omitted, and, possibly, decisions based on such analysis may not optimize overall welfare. In practice, as our analysis indicated inconsistent and incomplete inclusion of non-health consequences across CEAs, normative questions about how to define “relevant” non-health benefits and costs are often difficult to answer and may depend on the context. As Culyer et al. [21] noted, “the perspective will usually be context-dependent, with the context determining the appropriate perspective.”

Without knowing the context of the decision being made, researchers may continue to struggle to implement a broader societal perspective. A step forward would be to increase the use of an impact inventory, as this can ensure that all of the standardized consequences of interventions, including those falling outside the formal healthcare sector, are considered regularly and comprehensively [14]. Despite a low uptake of the impact inventory in the first 2 years since the Second Panel’s recommendation, the trend is upward (0.7% in 2017, 1.4% in 2018, and 2.7% in 2019 [based on partial 2019 data]), and the Institute for Clinical and Economic Review included it as part of its evidence assessments [45]. Widespread use of the impact inventory would provide the relevant information in a comprehensive, consistent, and transparent manner to guide decision makers with different preferences with regard to what should be included in a technology assessment. For example, a recent commentary provided a modified impact inventory to list a set of key health and social consequences to consider when evaluating the cost effectiveness of policy responses for coronavirus disease 2019 (COVID-19) [46].

Although relevant non-health consequences can be identified, how to measure and value these consequences poses challenges. Researchers have pointed to a few methodological questions involved in costing, such as whether estimated costs should explicitly account for lost productivity from morbidity, whether such effects are already captured in measuring QALYs, or whether analysts should capture unrelated downstream costs (e.g., future unrelated medical costs and non-health consumptions during the extended life-years) [47–52]. In addition, analytic difficulties—including the lack of available data (e.g., on informal caregiver time or non-labor market productivity) [53]—have impeded the widespread implementation of a societal perspective in practice. Developing a country-specific cost catalog for non-health consequences (e.g., criminal justice system resource use, hourly wages, and annual non-health consumption) can help standardize and promote CEAs from a societal perspective [54].

Our study documented the lack of inclusion of informal caregiver time (i.e., spillover costs). However, an important health consequence—spillover health effects on family members and informal caregivers of patients, which might be considered irrespective of the perspective chosen—is also often omitted [55]. For example, CEAs of interventions for Alzheimer’s disease or a pediatric population are more likely to include both types of spillover effects than the overall literature, but spillover health effects were considered substantially less often than spillover costs [56, 57]. Recent advances in methodology and applications for measuring and valuing spillover effects have provided a set of useful resources for analysts to incorporate the spillover effects into CEAs from both a healthcare sector and a societal perspective [58].

Our analyses have some limitations. The CEA and GHCEA registries catalog only English-language, published cost-per-QALY-gained and cost-per-DALY-averted studies and do not capture the gray literature (e.g., HTA reports that may not be disseminated in regularly published, indexed journals) or other databases. Still, one study found that our registry databases reached 95% of published cost-per-QALY or cost-per-DALY literature [59]. In addition, the classification of the analytic perspective and costing approach involves reviewer judgment based on the available information (e.g., whether the stated perspective was matched with cost components presented). Despite our efforts to reduce discrepancies by holding consensus meetings with at least two reviewers, others may render different judgments.

In the analysis of the national HTA guidelines, although we performed a detailed scoping literature search to identify the most recent country-specific recommendations, our search may have failed to identify updated versions of some of the recommendations. In addition, some of the guidelines provided scarce information about the recommended perspective. In these cases, we either used the perspective stated in the guideline or categorized the perspective based on the limited information provided.

## Conclusion

The choice of analytic perspective and cost components to include in a health economic evaluation can have an important impact on value assessment. Researchers and analysts should be transparent about their choices. Revised guidelines by the Second Panel, including the use of the impact inventory and reporting of disaggregated outcomes, can help reduce inconsistencies across analyses and the attendant confusion.

## Electronic supplementary material

Below is the link to the electronic supplementary material.

Supplementary file1 (DOCX 424 kb)

## Data Availability

The data used in our analysis are publicly accessible through Tufts Medical Center’s CEA registry (restricted public access), the GHCEA registry (open access), and country-specific guidelines (available online). The specific analytic datasets are available from the corresponding author upon reasonable request.
