# Correction to: Perspective and Costing in Cost‑Effectiveness Analysis, 1974–2018

**DOI:** 10.1007/s40273-020-00968-6

**Published:** 2020-10-21

**Authors:** David D. Kim, Madison C. Silver, Natalia Kunst, Joshua T. Cohen, Daniel A. Ollendorf, Peter J. Neumann

**Affiliations:** 1 Center for the Evaluation of Value and Risk in Health, Institute for Clinical Research and Health Policy Studies, Tufts Medical Center, 800 Washington St, Box 063, Boston, MA 02111 USA; 2 Department of Medicine, Tufts University School of Medicine, Boston, MA USA; 3 Department of Health Management and Health Economics, University of Oslo, Oslo, Norway; 4 Cancer Outcomes, Public Policy and Effectiveness Research (COPPER) Center, Yale University School of Medicine, New Haven, CT USA; 5 LINK Medical Research, Oslo, Norway

## Correction to: PharmacoEconomics (2020) 38:1135–1145 10.1007/s40273-020-00942-2

The article Perspective and Costing in Cost-Effectiveness Analysis, 1974–2018, written by David D. Kim· Madison C. Silver. Natalia Kunst . Joshua T. Cohen. Daniel A. Ollendorf . Peter J. Neumann was published under the incorrect Creative Commons (CC) license (CC-BY-NC). The correct license is CC-BY. Open access for this paper was funded by Bill and Melinda Gates Foundation, Inc. This article is licensed under a Creative Commons Attribution 4.0 International License, which permits use, sharing, adaptation, distribution and reproduction in any medium or format, as long as you give appropriate credit to the original author(s) and the source, provide a link to the Creative Commons license, and indicate if changes were made. The images or other third party material in this article are included in the article’s Creative Commons license, unless indicated otherwise in a credit line to the material. If material is not included in the article’s Creative Commons license and your intended use is not permitted by statutory regulation or exceeds the permitted use, you will need to obtain permission directly from the copyright holder. To view a copy of this license, visit https://creativecommons.org/licenses/by/4.0/.

The original article has been corrected.

