# Supplementary material for: Perspective and Costing in Cost-Effectiveness Analysis, 1974–2018
Source: Pharmacoeconomics. 2020 Oct 20;38(10):1135–45. doi: 10.1007/s40273-020-00942-2 (PMC7373843; doi:10.1007/s40273-020-00942-2)
Supplement: Supplementary file 1 — Supplementary file1 (DOCX 424 kb) [file 40273_2020_942_MOESM1_ESM.docx]

**Online Supplements A**

**Table A. Change over time in perspective used in published cost-effectiveness analyses between 1974 and 2018**

|  | **1974-1999** | **2000-2009** | **2010-2014** | **2015-2018** | **P-value** |
| --- | --- | --- | --- | --- | --- |
| **Cost-per-QALY gained: 1974-2018 (N=6,907)** | | | | | |
|  | (n= 346) | (n=2,088) | (n= 2,403) | (n= 2,070) |  |
| Societal / Limited Societal | 24.9% | 27.8% | 22.2% | 21.5% | <.01 |
| Health Care Sector / Payer | 73.7% | 70.1% | 76.3% | 78.0% | <.01 |
| Not stated/could not be determined^†^ | 0.9% | 1.4% | 0.8% | 0.4% | 0.01 |
| Other | 0.6% | 0.8% | 0.7% | 0.0% | <.01 |
| **Cost-per-DALY gained: 1995-2018 (N=698)** | | | | | |
|  | (n=13) | (n=170) | (n=236) | (n=279) |  |
| Societal / Limited Societal | 38.5% | 29.8% | 30.1% | 33.2% | 0.774 |
| Health Care Sector / Payer | 61.5% | 69.0% | 67.8% | 62.2% | 0.406 |
| Not stated/could not be determined^†^ | 0.0% | 0.6% | 0.8% | 2.5% | 0.284 |
| Other | 0.0% | 0.6% | 1.3% | 2.1% | 0.565 |

† Authors did not provide sufficient information to determine the types of costs or benefits evaluated.

**Table B. Cost-effectiveness and inclusion of cost components by study type**

|  | **Number of ratios (% of total)** | **Number of Cost-saving (% of total)** | **Median ICER** | **IQR** | **Number of Dominated (% of total)** |
| --- | --- | --- | --- | --- | --- |
| ***Cost-per-QALY gained (1974-2018)*** | | | | | |
| **All ratios** | **19,946** | **3,675 of 19,946** | **26,000** | **(2,900-110,000)** | **1,813 of 19,946** |
|  | **(100%)** | **(18%)** |  |  | **(9%)** |
| **Health Care Sector Perspective** | 15,156 | 2,744 of 15,156 | 25,000 | (3,100-100,000) | 1,280 of 15,156 |
|  | (76%) | (17%) |  |  | (8%) |
| **Intervention^a^** |  |  |  |  |  |
| Care delivery | 1,110 (7%) | 234 | 19,000 | (1,300-79,000) | 66 |
| Health Education or Behavior | 864 (6%) | 194 | 9,400 | (295-40,000) | 46 |
| Immunization | 710 (5%) | 87 | 25,500 | (4,600-79,00) | 18 |
| Medical / Surgical Procedure | 3,132 (21%) | 609 | 21,000 | (2,400-120,000) | 319 |
| Medical Device | 943 (6%) | 188 | 30,000 | (3,100-91,000) | 72 |
| Other | 544 (4%) | 114 | 21,000 | (1,900-67,500) | 41 |
| Pharmaceutical | 8,207(54%) | 1,597 | 25,000 | (2,900-110,000) | 721 |
| Screening / Diagnostic | 3,350 (22%) | 490 | 34,000 | (5,400-140,000) | 337 |
| **Societal Perspective** | 4,455 | 900 of 4,455 | 30,000 | (2,300-150,000) | 508 of 4,455 |
|  | (22%) | (20%) |  |  | (10%) |
| **Intervention^a^** |  |  |  |  |  |
| Care delivery | 462 (10%) | 101 | 28,000 | (1,700-100,000) | 29 |
| Health Education or Behavior | 562 (13%) | 144 | 9,400 | (295-40,000) | 56 |
| Immunization | 62 (14%) | 130 | 14,000 | (1,100-82,000) | 29 |
| Medical / Surgical Procedure | 704 (16%) | 122 | 33,000 | (3,450-180,000) | 95 |
| Medical Device | 201 (5%) | 49 | 27,000 | (430-140,000) | 35 |
| Other | 260 (6%) | 73 | 14,500 | (Dominant-67,500) | 16 |
| Pharmaceutical | 1,518 (34%) | 331 | 29,000 | (1,000-160,000) | 93 |
| Screening / Diagnostic | 1,208(27%) | 103 | 59,500 | (9,950-565,000) | 226 |
| ***Cost-per-DALY averted (1995-2018)*** | | | | | |
| **All ratios** | **5,572** | **454 of 5,572** | **430** | **(67-3,400)** | **161 of 5,572** |
|  | **(100%)** | **(8%)** |  |  | **(3%)** |
| **Health Care Sector Perspective** | 4,147 | 291 of 4,147 | 460 | (74-3500) | 140 |
|  | (74%) | (7%) |  |  | (3%) |
| **Intervention^a^** |  |  |  |  |  |
| Care Delivery | 734 (18%) | 45 | 2,850 | (160-11,000) | 44 |
| Health Education or Behavior | 1,167 (28%) | 65 | 720 | (110-3,300) | 9 |
| Immunization | 1,040 (25%) | 94 | 270 | (50-1,400) | 47 |
| Medical / Surgical Procedure | 408 (10%) | 12 | 390 | (74-1,900) | 2 |
| Medical Device | 77 (2%) | 8 | 430 | (17-Dominated) | 30 |
| Others | 278 (7) | 5 | 180 | (29-6300) | 3 |
| Pharmaceutical | 1,740 (42%) | 100 | 1,300 | (120-5,350) | 19 |
| Screening / Diagnostic | 976 (24%) | 32 | 860 | (160-6,200) | 72 |
| Maternal/Neonatal | 324 (8%) | 15 | 61 | (7-395) | 2 |
| **Societal Perspective** | 1,274 | 159 of 1,274 | 345 | (51-2,700) | 21 of 1,274 |
|  | (23%) | (12%) |  |  | (2%) |
| **Intervention^a^** |  |  |  |  |  |
| Care Delivery | 177 (14%) | 13 | 1,200 | (240-5,300) | 1 |
| Health Education or Behavior | 272 (23%) | 12 | 720 | (110-3,300) | 4 |
| Immunization | 606 (48%) | 88 | 390 | (87-2,800) | 4 |
| Medical / Surgical Procedure | 217 (19%) | 2 | 1,500 | (410-6,600) | 5 |
| Medical Device | 28 (2%) | 0 | 4,450 | (715-39,000) | 1 |
| Others | 65 (5%) | 4 | 150 | (17-1,400) | 0 |
| Pharmaceutical | 366 (29%) | 29 | 485 | (52-3,500) | 11 |
| Screening / Diagnostic | 343 (27%) | 54 | 480 | (90-3,500) | 343 |
| Maternal/Neonatal | 36 (3%) | 2 | 95 | (11-385) | 4 |

**^a^** Intervention types are not mutually exclusive categories.

**Table C. Analysis of incremental cost-per-QALY ratios by perspectives and study sponsor**

| Intervention Types | Number of ratios | Number of cost-saving | Median ICER | IQR | Number of Dominated |
| --- | --- | --- | --- | --- | --- |
|  |  | (% of total) |  |  | (% of total) |
| **Health Care Sector perspective** | |  |  |  |  |
| All | 15,156 (100%) | 2,744 of 15,156 (18.1%) | 25,000 | (3,100 -100,000) | 1,280 of 15,156 (8.5%) |
| Non-industry sponsored | 10,383 (69%) | 1,629 of 10,383 (15.7%) | 31,000 | (4,000-140,000) | 992 of 10,383 (9.6%) |
| Industry-sponsored | 4,773 (31%) | 1,115 of 4,773 (23.3%) | 18,000 | (560 -54,000) | 288 of 4,773 (6.0%) |
| **Societal perspective** |  |  |  |  |  |
| All | 4,455 (100%) | 900 of 4,455 (20.2%) | 30,000 | (2,300-150,000) | 508 of 4,455 (11.4%) |
| Non-industry sponsored | 3,550 (80%) | 629 of 3,550 (17.7%) | 36,000 | (3,900-200,000) | 449 of 3,550 (12.7%) |
| Industry-sponsored | 905 (20%) | 271 of 905 (29.9%) | 27,000 | (Cost-saving-63,000) | 59 of 905 (6.5%) |

Source: Author's analysis of CEA Registry (www.cearegistry.org)

**Table D. Analysis of incremental cost-per-DALY ratios by perspectives and study sponsor**

| Intervention Types | Number of ratios  (%) | Number of cost-saving | Median ICER | IQR | Number of Dominated |
| --- | --- | --- | --- | --- | --- |
|  |  | (% of total) |  |  | (% of total) |
| **Health Care Sector perspective** | |  |  |  |  |
| All | 4,147 (100%) | 291 of 4,147 (7.0%) | 460 | (74 -3,500) | 140 of 4,147 (3.4%) |
| Non-industry sponsored | 4,039 (97%) | 280 of 4,039 (6.9%) | 470 | (81-3,700) | 140 of 4,039 (3.5%) |
| Industry-sponsored | 108 (3%) | 11 of 108 (10.2%) | 24 | (7 -650) | 0 of 108 (0%) |
| **Societal perspective** |  |  |  |  |  |
| All | 1,274 (100%) | 159 of 1,274 (12.5%) | 345 | (51-2,700) | 21 of 1,274 (1.7%) |
| Non-industry sponsored | 1,185 (93%) | 149 of 1,185 (12.6%) | 310 | (50-2,400) | 21 of 1,185 (1.8%) |
| Industry-sponsored | 89 (7%) | 10 of 89 (11.2%) | 2,000 | (210-4,300) | 0 of 89 (0%) |

Source: Author's analysis of the Global Health CEA Registry (www.ghcearegistry.org)

**Table E. A list of the reviewed 45 countries and their recommended perspectives based on national guidelines on health technology assessment**^†^

| **Recommended perspectives**  **(N=45)** | **Countries  (Year of Most Recent Guidelines Identified)** |
| --- | --- |
| Payer perspective only  (n=3) | Israel (2010), New Zealand (2015), Scotland (2017) |
| Payer + a supplementary societal or other perspective  (n=18) | Argentina (2015), Brazil (2015), Canada (2017), Chile (2013), Colombia (2014), Croatia (2011), Czech Republic (2017), England & Wales (2013), Hungary (2017), Malaysia (2012), Mexico (2015), Ireland (2019), Japan (2019), Paraguay (2015), Slovak Republic (2012), Slovenia (2013), South Africa (2013), Uruguay (2015) |
| Health care sector perspective only  (n=4) | Belgium (2012), Germany (2009), Norway (2018) ^‡^, Singapore (2019) |
| Health care sector + a supplementary societal or other perspective (n=6) | Australia (2016), Egypt (2013), Estonia (2002), Latvia (2002), Lithuania (2002), Poland (2016) |
| Societal perspective only  (n=8) | Cuba (2003), Denmark (2008), Finland (2017), France (2012), Portugal (1998), South Korea (2013), Sweden (2017), Thailand (2017) |
| Societal perspective + a supplementary payer or other perspective  (n=3) | Austria (2006), Netherland (2016), Taiwan (2009) |
| Both societal and payer perspectives  (n=3) | Indonesia (2017), Italy (2001), Spain (2010) |

† Full review summary of these guidelines is available in the Online Supplement B.
‡Norway recommends an extended health care sector perspective.

**Figure A. Trends in analytic perspectives used in cost-per-DALY studies: 1995-2018 (N=698)**


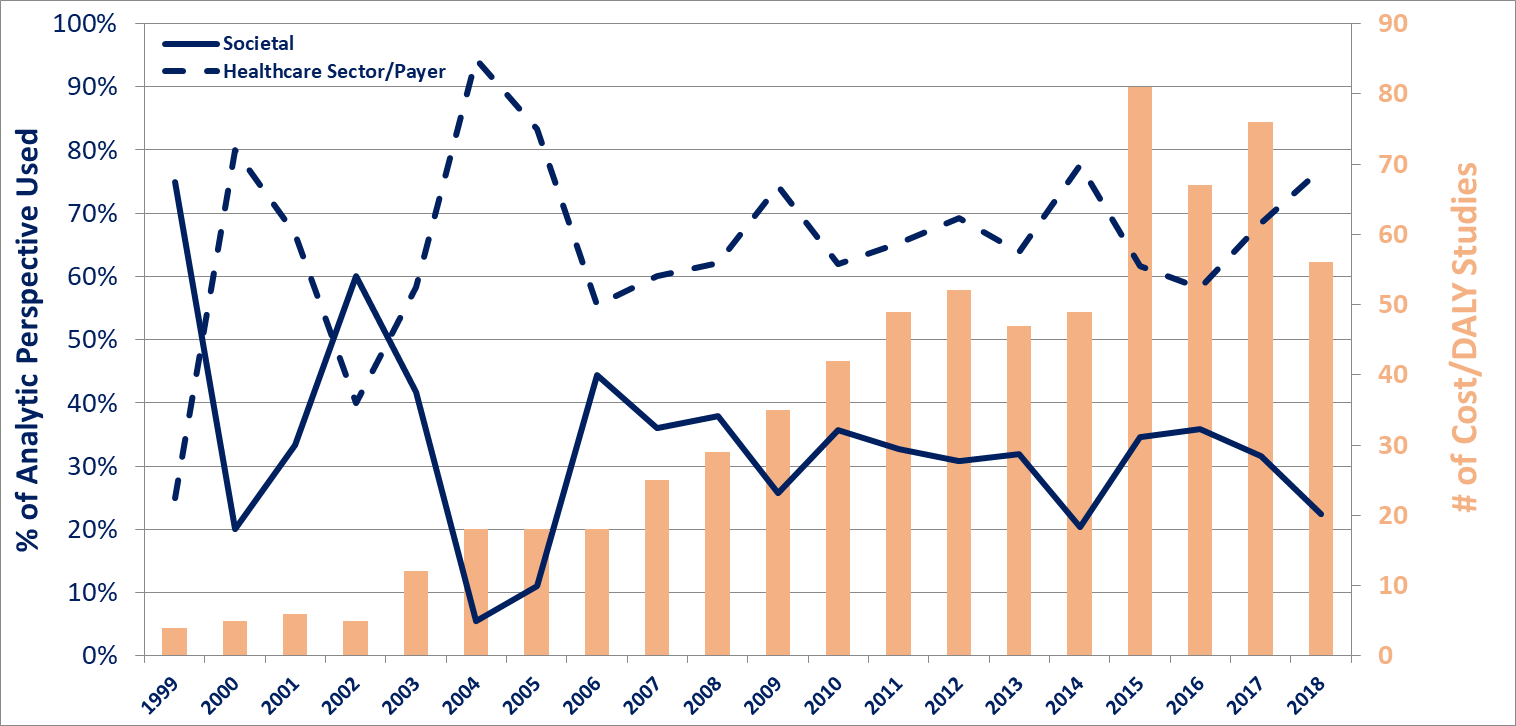
Note: With a relatively small number of cost-per-DALY studies published prior to 1999 (n=9, 1.3%), the Figure shows the data points
since 1999.

**Figure B. Incremental cost-effectiveness ratios by perspective used and intervention types
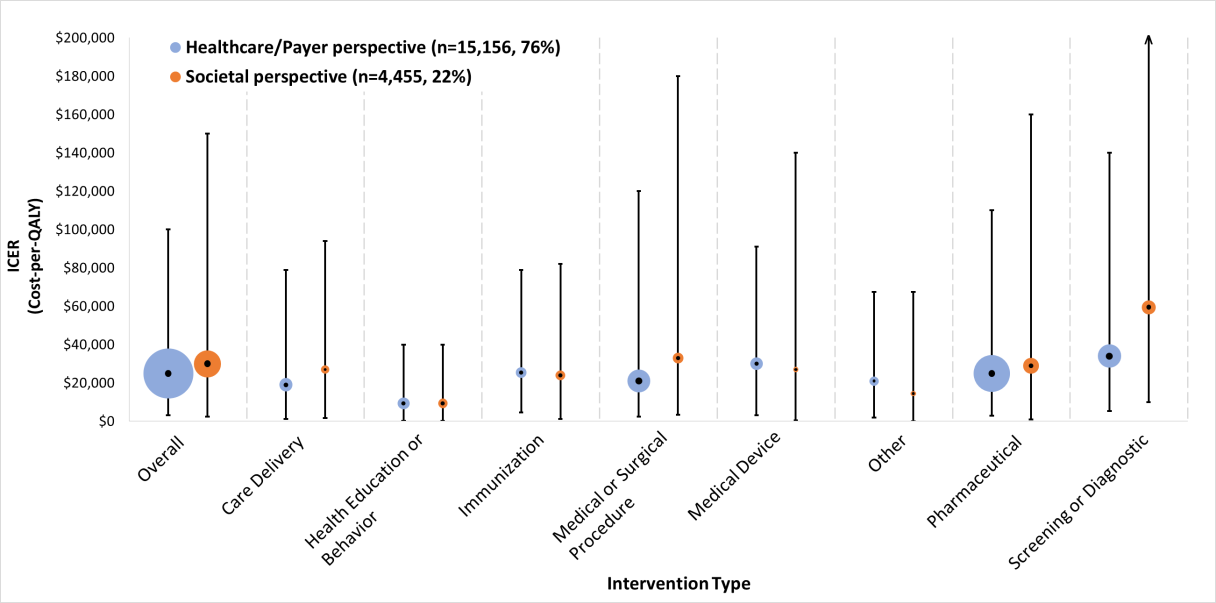


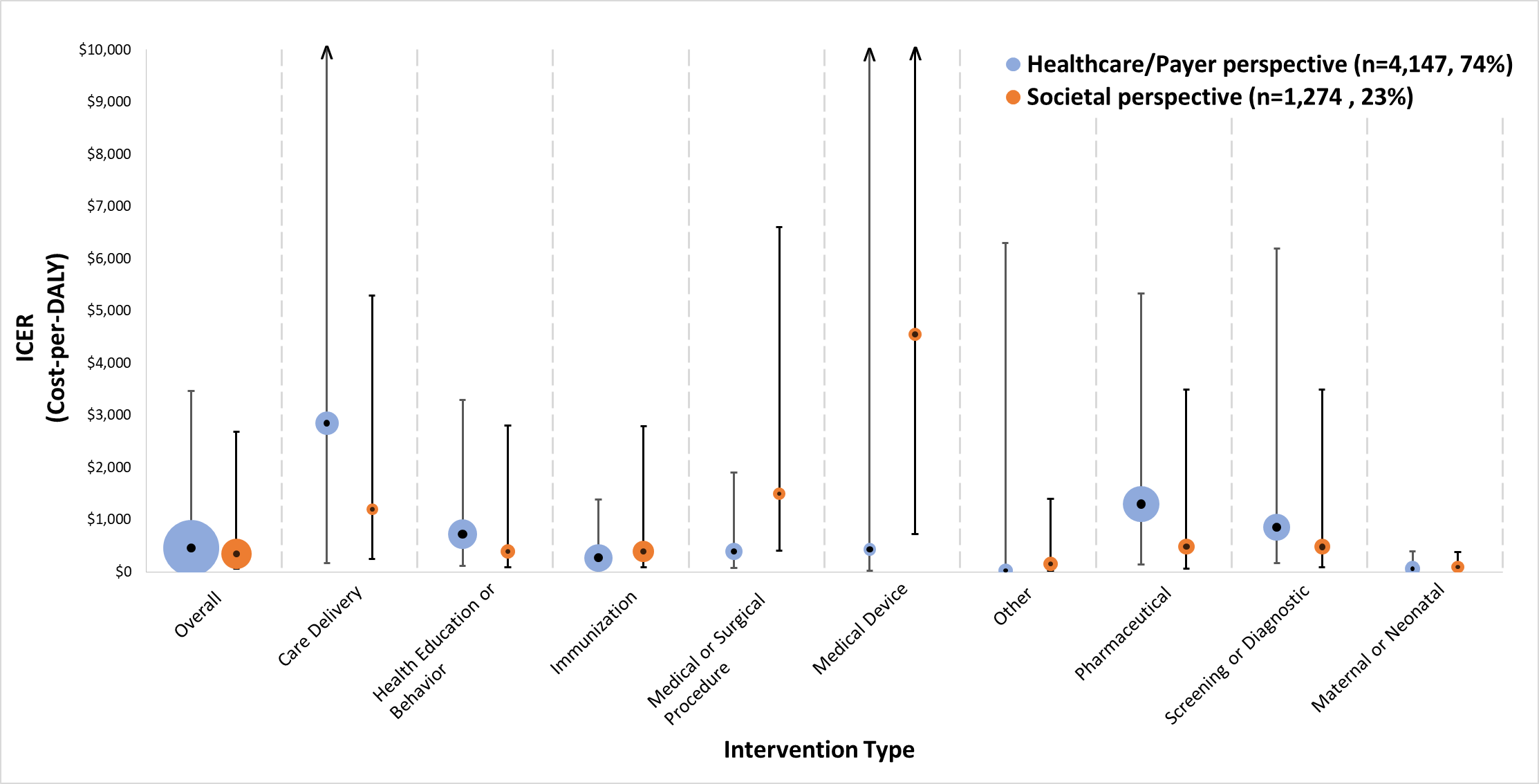
**NOTE: The size of the circle represents the volume of included studies for each perspective and intervention type. The center of the circle denotes the median incremental cost-effectiveness ratios (ICER) while the lines extend to the 25^th^ and 75^th^ percentiles, the inter-quartile range (IQR). Due to the wide IQR of some ICERs, we used an arrow to represent ICERs that were greater than the maximum of the y-axis at the 75th percentile. Lines extending to the x-axis represent interventions that were cost-saving at the 25th percentile

**Figure C. The country-specific use of the societal perspective

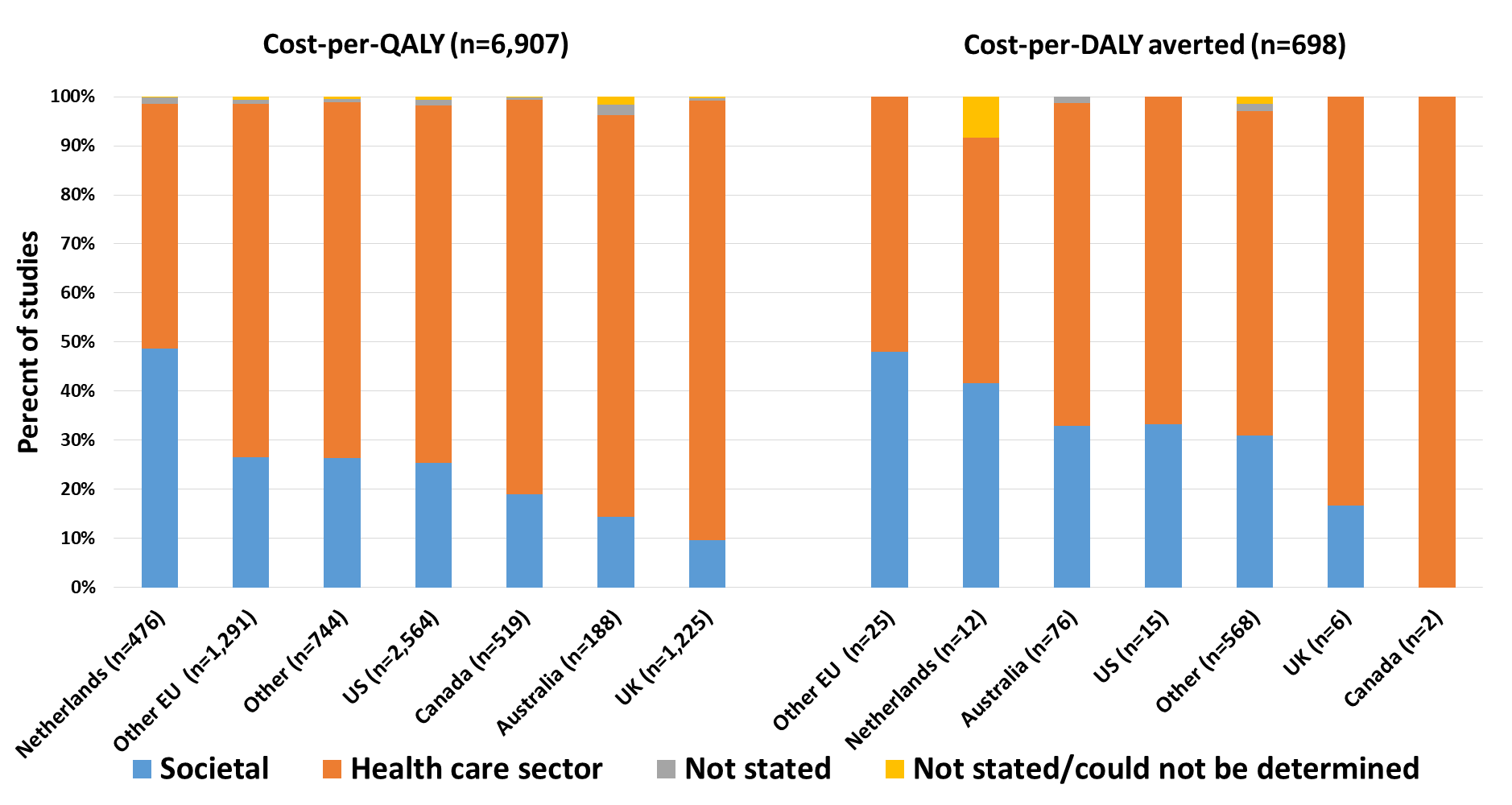
**

Note: Among the top 5 CEA producing countries (the United States, the United Kingdom, Canada, Netherlands, and Australia), Netherlands is the only country recommending a societal perspective while the UK, Canada, and Australia recommend a narrower (national payer or health care sector) perspective. (Despite several guidelines and the existence of the Institute for Clinical and Economic Review, the US does not have any governing body to provide national guidelines on health technology assessment.

**Online Supplement B: The Summary of 45 country-specific guidelines**

| **Country** | **Perspective** | **Guideline** | **Description** |
| --- | --- | --- | --- |
| Australia  2016 | Health care sector perspective with a supplementary societal or other perspective | “Guidelines for preparing a submission to the Pharmaceutical Benefits Advisory Committee (Version 5.0)”  Pharmaceutical Benefits Advisory Committee. Australian Government Department of Health  <https://pbac.pbs.gov.au/content/information/files/pbac-guidelines-version-5.pdf>  2016 | “Use a health care system perspective to inform the base-case analysis, and describe any alternative perspectives provided as supplementary analyses (Subsection 3A.1.4). […] Section 3 adopts a broad perspective for the valuation of health care resources, so include all contributions to the costs of health care resources – including those paid for by patients, governments, health insurance agencies and any other part of society – in the economic evaluation.” |
| Austria  2006 | Societal perspective with a supplementary payer or other perspective | “Guidelines on Health Economic Evaluation. Consensus paper”  Institute for Pharmaeconomic Research (IPF)  [http://www.ipf-ac.at/fileadmin/template/PDF alt/Konsens_Guidelines_en.pdf](http://www.ipf-ac.at/fileadmin/template/PDF%20alt/Konsens_Guidelines_en.pdf) | “Apart from the societal/economic perspective, which represents the most comprehensive approach, other perspectives are possible, e.g. the health system, social insurance, other service providers (hospitals), etc. The choice of perspective must be justified. If several perspectives are included in the analysis, the results must be presented separately for each study perspective.” |
| Baltic: Latvia, Lithuania, Estonia  2002 | Health care sector perspective with a supplementary societal or other perspective | “Baltic guideline for economic evaluation of pharmaceuticals”  Medicines’ Pricing and Reimbursement Agency, Latvia  Health Insurance Fund, Estonia  Department of Pharmacy under the Ministry of Health, Lithuania  <https://tools.ispor.org/PEguidelines/source/Baltic-PE-guideline.pdf> | “All analyses are to be conducted principally from a health care perspective (including only direct health care costs and benefits for healthcare). Analyses from a societal perspective (including all costs and benefits outside the healthcare system) may only be presented in addition, if considered relevant by the applicant.” |
| Belgium  2012 | Health care sector perspective only | “Belgian Guidelines for Economic Evaluations and Budget Impact Analyses: Second Edition”  Belgian Health Care Knowledge Centre (KCE)  <https://kce.fgov.be/sites/default/files/atoms/files/KCE_183_economic_evaluations_second_edition_Report_update.pdf> | “In conclusion, the base-case analysis should be performed from the perspective of the health care payers (federal government + communities + patients). Analyses from a broader perspective are allowed but should be clearly distinguished from the reference case.” |
| Canada  2017 | Payer perspective with a supplementary societal or other perspective | “Guidelines for the Economic Evaluation of Health Technologies: Canada — 4th Edition”  Canadian Agency for Drugs and Technologies in Health (CADTH)  <https://www.cadth.ca/dv/guidelines-economic-evaluation-health-technologies-canada-4th-edition> | “In the reference case, the publicly funded health care payer perspective should be adopted (see Appendix 2) and the included costs should be those incurred by the Canadian public payer, and the included outcomes should reflect all meaningful health effects for patients and their informal caregivers. […]Perspectives other than that considered in the reference case analysis may also be of interest if they are expected to have an impact on the results.” |
| Chile  2013 | Payer perspective with a supplementary societal or other perspective | “Guía Metodológica para la Evaluación Económica de Intervenciones en Salud en Chile”  Departamento de Economía de la Salud Subsecretaría de Salud Pública Ministerio de Salud de Chile  <http://www.orasconhu.org/case/sites/default/files/files/EE_FINAL_web.pdf> | “Recomendación sobre perspectiva: En el caso de los estudios en que el mandante sea el Ministerio de Salud, la perspectiva para el caso de referencia es la del sector público de salud. La incorporación de la perspectiva del paciente se recomienda cuando las alternativas siendo evaluadas afectan de manera distinta el uso de recursos del paciente y su familia. En todo caso cuando se incluyan otras perspectivas en el análisis, los resultados deben ser presentados por separado a los del caso de referencia (cuya perspectiva es sector público de salud).”  Translation:  *Perspective Recommendation: In the case of studies in which the principal is the Ministry of Health, the perspective for the reference case is that of the public health sector. The incorporation of the patient's perspective is recommended when the alternatives being evaluated affect the use of resources of the patient and his family differently. In any case, when other perspectives are included in the analysis, the results must be presented separately to those of the reference case (whose perspective is the public health sector).* |
| Colombia  2014 | Payer perspective with a supplementary societal or other perspective | “Manual para la elaboración de evaluaciones económicas en salud”  Instituto de Evaluación Tecnológica en Salud – IETS  <http://www.iets.org.co/Archivos/64/Manual_evaluacion_economica.pdf> | “Para el caso de referencia colombiano, se recomienda la perspectiva del sistema de salud colombiano, que para efectos de este manual implica incluir el valor de todos los recursos directos asociados al uso de la tecnología objeto de la evaluación y los beneficios en salud percibidos directamente por los pacientes, sin incluir cambios en la productividad o costos o beneficios en otros sectores de la sociedad. Tampoco serán incluidos gastos de bolsillo, entendidos estos como gastos de transporte del paciente o de los cuidadores, cuotas moderadoras o copagos.”  Translation:  *“For the Colombian reference case, the Colombian health system perspective is recommended, which for the purposes of this manual implies including the value of all direct resources associated with the use of the technology under evaluation and the health benefits directly perceived by patients, not including changes in productivity or costs or benefits in other sectors of society. Neither will be included out-of-pocket expenses, understood as transportation expenses for the patient or caregivers, moderating fees or co-payments.”* |
| Cuba  2003 | Societal perspective only | “Guía metodológica para la evaluación económica en salud. Cuba, 2003”  Ministerio de Salud Pública. Escuela Nacional de Salud Pública  <http://scielo.sld.cu/scielo.php?script=sci_arttext&pid=S0864-34662004000100005> | “Los estudios deben realizarse desde la perspectiva social y desglosarse en otros puntos de vista que tengan relevancia en el estudio.”  Translation:  *“Studies should be carried out from a social perspective and broken down into other points of view that are relevant in the study.”* |
| Croatia  2011 | Payer perspective with a supplementary societal or other perspective | “The Croatian Guideline for Health Technology Assessment Process and Reporting”  Agency for Quality and Accreditation in Health Care, Croatia  Department for Development, Research and Health Technology Assessment  <http://aaz.hr/sites/default/files/hrvatske_smjernice_za_procjenu_zdravstvenih_tehnologija.pdf> | “Perspective on costs: Croatian Institute for Health Insurance (Croatian Institute for Health Insurance as public payer) (societal perspective, including all cost and benefits outside the health care system, may be presented in addition, if considered relevant for some topics). […] This does not preclude additional analyses being presented when one or more aspects of methods differ from the reference case. However, these must be justified and clearly distinguished from the reference case. For example, analyses from a societal perspective (including all cost and benefits outside the health care system) may be presented in addition to reference case ones, if considered relevant for some topics.” |
| Czech Republic  2017 | Payer perspective with a supplementary societal or other perspective | “Postup pro posuzování analýzy nákladové efektivity”  Státní Ústav pro Kontrolu Léčiv  <http://www.sukl.cz/file/85788_1_1> | “6.10. Perspektiva hodnocení. Definice a požadavek: Pro potřeby prokázání nákladové efektivity podle ustanovení § 15 odst. 8 zákona o veřejném zdra- votním pojištění je přípustná pouze perspektiva zdravotních pojišťoven ČR (plátce za vykázanou zdravotní péči, payer). Ostatní náklady mohou být informativně vyčísleny, ale musejí být vyjádřeny zcela odděleně. Posouzení: Hodnotitel zkontroluje, zda byla zvolena perspektiva plátce za zdravotní péči a zda zahrnuté náklady a přínosy této perspektivě odpovídají. Zvolení jiné perspektivy nebo jasné oddělení nerelevantních (např. nepřímých) nákladů činí farmakoekonomické hodnocení nesprávně provedeným.”  Translation:  *“6.10. Perspective of evaluation. Definition and requirement:*  *For the purposes of proving cost-effectiveness pursuant to Section 15 (8) of the Public Health Insurance Act, only the perspective of the Czech health insurance companies (payer for reported health care, payer) is permissible. Other costs can be quantified in an informative manner, but must be expressed separately.”* |
| Denmark  2008 | Societal perspective only | “Health Technology Assessment Handbook”  Danish Centre for Health Technology Assessment, National Board of Health  <https://www.sst.dk/~/media/ECAAC5AA1D6943BEAC96907E03023E22.ashx> | “Danish and Canadian guidelines also recommend that analyses should be performed with a societal perspective (5,17) to avoid such situations. If one wants to perform an economic analysis with a narrower perspective, *there must be good arguments for this*. These might be, for example, that the inclusion of other costs would merely confirm the result achieved with a narrower perspective.” |
| Egypt  2013 | Health care sector perspective with a supplementary societal or other perspective | “Recommendations for Reporting Pharmacoeconomic Evaluations in Egypt”  Ministry of Health and Population (MOHP) in Egypt. Pharmacoeconomic Unit  [https://www.sciencedirect.com/science/article/pii/S2212109913000770?via=ihub - bib17](https://www.sciencedirect.com/science/article/pii/S2212109913000770?via%3Dihub#bib17) | “Resource use and costs should be identified, measured in their natural units and values [17]. The primary perspective for these studies is the overall health care services. Therefore, the resources that should be considered are direct medical costs, which include drugs, medical devices, medical services including procedures, laboratory, or diagnostic tests, hospital services and emergency department visits, and primary care visits. Other direct nonmedical and indirect costs paid by patients, including lost productivity costs, might be included only in the sensitivity analysis. If indirect costs are included in the analysis, the rationality of the costs and how they are estimated should be explained. Current and future costs arising as a consequence of a product, and occurring during the specified time frame of the study, should also be included. Mean values should be used. Different costs or costs of the same resources that are used in different quantities should be included in the analysis [18].” |
| England & Wales  2013 | Payer perspective with a supplementary societal or other perspective | “Guide to the methods of technology appraisal 2013”  National Institute for Health and Care Excellence (NICE)  <https://www.nice.org.uk/process/pmg9/chapter/foreword>  <https://www.nice.org.uk/process/pmg20/chapter/incorporating-economic-evaluation>  “Perspective”  York Health Economics Consortium  <https://yhec.co.uk/glossary/perspective/> | “2.2.9 The potential impact on resource costs and savings that would be expected from the introduction of the technology should be considered from the perspective of the NHS and personal social services. In exceptional circumstances, when requested by the Department of Health in the remit for the appraisal, the scope will list requirements for adopting a broader perspective on costs. Perspective 5.1.7 For the reference case, the perspective on outcomes should be all direct health effects, whether for patients or other people. The perspective adopted on costs should be that of the NHS and personal and social services. 5.1.8 The reference-case perspective on outcomes aims to maximise health gain from available healthcare resources. Some features of healthcare delivery often referred to as 'process characteristics' may ultimately have health consequences, for example, mode of treatment delivery through its impact on adherence. If characteristics of healthcare technologies have a value to people independent of any direct effect on health, the nature of these characteristics should be clearly explained and if possible the value of the additional benefit should be quantified. These characteristics may include convenience and the level of information available for patients. 5.1.9 The Institute does not set the budget for the NHS. The appropriate objective of the Institute's technology appraisal programme is to offer guidance that represents an efficient use of available NHS and personal social services resources. For these reasons, the reference-case perspective on costs is that of the NHS and personal social services.” “In its reference case UK NICE recommends a perspective of ‘NHS and personal and social services’, recognising that the societal perspective may bias against those not in work, such as people over retirement age or those not able to work due to health reasons. The NHS perspective includes treatment costs such as medicine costs, administration and monitoring, other health service resource use costs associated with the managing the disease (e.g. GP visits, hospital admissions), and costs of managing adverse events caused by treatment. It does not include patients’ costs of obtaining care such as transportation, over the-counter purchases, co-payments or time off work.” |
| Finland  2017 | Societal perspective only | “Preparing a Health Economic Evaluation to be Attached to the Application for Reimbursement Status and Wholesale Price for a Medicinal Product”  Finnish Medicines Agency Fimea  <http://www.hila.fi/c/document_library/get_file?folderId=1133981&name=DLFE-9844.pdf> | “The calculation of costs must include, irrespective of the payer, all direct health care and comparable social welfare costs related to the therapies that are being compared. An examination of the costs of medicinal products alone is not sufficient, except for situations where the cost of the medicinal products is the only difference between the treatments. If productivity losses are included in the cost calculation, the results must also be presented so that those are excluded. A detailed account must be presented of the resources used and unit costs, giving the grounds and source references. The health economic evaluation must be based on as up-to-date information on the costs in Finland as possible.” |
| France  2012 | Societal perspective only | “Choices in Methods for Economic  Evaluation”  Department of Economics and Public Health Assessment, Haute Autorité de Santé (HAS)  <https://www.has-sante.fr/upload/docs/application/pdf/2012-10/choices_in_methods_for_economic_evaluation.pdf> | In the context of HAS and its work, the aim of the economic evaluation is to shed light on public decision-making in the allocation of resources.  The reference case analysis adopts a collective perspective. This allows account to be taken of all stakeholders affected by the decision, either because they are affected by one of the health consequences of the health interventions, or because they are involved as healthcare funders.  The economic evaluation covers the costs and health effects of interventions under real conditions of use, whether observed or expected. The evaluation of costs endeavours to identify, measure and value all the resources consumed in the production of the interventions studied, whatever the source of funding (patients, compulsory and supplementary health insurance schemes, the central government, etc.)  Evaluation of the health outcomes identifies the health effects relevant from the point of view of the individuals concerned (see below). These are then measured in life years, possibly weighted by a valuation of HRQL (preference-based scores). In this case, preference-based scores are obtained from a representative sample of the general population (see the section on cost-utility evaluation, page 28). |
| Germany  2009 | Health care sector perspective only | “Working Paper Modelling Institute for Quality and Efficiency in Health Care”  Institute for Quality and Efficiency in Health Care (IQWiG)  <https://www.google.com/url?sa=t&rct=j&q=&esrc=s&source=web&cd=1&ved=2ahUKEwiFju-1tsPnAhWEhOAKHZn8CtkQFjAAegQIARAB&url=https%3A%2F%2Fwww.iqwig.de%2Fdownload%2FWorking_Paper_Modelling.pdf&usg=AOvVaw37_FkNIs9o8sc0cDLVYekE> | “7.2 Perspective: The primary perspective of the analysis should be that of the community of Statutory Health Insurance (SHI) insurants. This is not identical to the perspective of the SHI itself, as it includes resources of patients. If possible, cost results should be reported in aggregated as well as in completely disaggregated form. A comprehensive description of cost components is provided in the working paper “Cost Estimation”.” |
| Hungary  2017 | Payer perspective with a supplementary societal or other perspective | “Professional Healthcare Guideline on the Methodology of Health Technology Assessment”  National Institute of Pharmacy and Nutrition  <https://tools.ispor.org/PEguidelines/source/HTA_Guideline_HUN_eng.pdf> | “As a primary choice, the payer perspective is recommended. Besides that, if possible, a comprehensive societal perspective should be aimed for, depending on the given technology.” |
| Indonesia  2017 | Both societal and payer perspectives | “Health Technology Assessment (HTA) Guideline.”  Indonesian Health Technology Assessment Committee (InaHTAC)  Ministry of Health, Indonesia  <http://adphealth.org/upload/resource/FINAL_HTA_ENG_-1.pdf> | “HTA in Indonesia uses both societal and provider perspectives.” |
| Ireland  2019 | Payer perspective with a supplementary societal or other perspective | “Guidelines for the Economic Evaluation of Health Technologies in Ireland”  Health Information and Quality Authority  <https://www.hiqa.ie/sites/default/files/2019-07/HTA-Economic-Guidelines-2019.pdf> | “Study perspective (Section 2.3) For the reference case, the perspective of the publicly-funded health and social care system in Ireland should be adopted when assessing costs. […] However, limiting the perspective of a study to that of the primary stakeholders in the healthcare system may lead to healthcare policies that fail to optimise efficiency and social benefit. Adopting a societal perspective that captures all relevant costs and consequences of the technologies in question, regardless on who these costs and consequences fall, is considered the most use of the finite resources available to the Health Service Executive (HSE). […] In some circumstances, it may be appropriate to provide a secondary analysis that is not a full societal perspective but extends beyond the HSE and Department of Health to include other relevant government departments. For example, if there are significant costs or savings accruing to departments other than health (for example, the Department of Education). Inclusion of such an analysis must be clearly justified and supported by sufficient evidence.” |
| Israel  2010 | Payer perspective only | “Guidelines for the submission of a request to include a pharmaceutical product in the national list of health services”  Ministry of Health Pharmaceutical Administration  <https://tools.ispor.org/PEguidelines/source/Israel-Guidelines-for-submission_2010.pdf> | “The perspective adopted on costs is that of the supplier (Ministry of Health, Health Management Organizations). The perspective adopted on benefits is of the health care system.” |
| Italy  2001 | Both societal and payer perspectives | “Guidelines for Economic Evaluations in Italy: Recommendations from The Italian Group of Pharmacoeconomic Studies”  Italian Group for Pharmacoeconomic Studies  <https://link.springer.com/article/10.1177/009286150103500122> | “The analysis must be carried out at least from society’s and the Italian National Health Service points of view. Other points of view (such as medical structure, patient, etc.) should be considered a useful sub-analysis.” |
| Japan  2019 | Payer perspective with a supplementary societal or other perspective | “Guideline for Preparing  Cost-Effectiveness Evaluation  to the Central Social Insurance  Medical Council”  National Institute of Public Health (C2H)  <https://c2h.niph.go.jp/tools/guideline/guideline_en.pdf>  <https://tools.ispor.org/PEguidelines/source/Japanese_PE_Guideline.pdf> | “2 Analysis perspective. 2.1 The perspective of the analysis should be specified. In particular, the analysis should consider the range of costs corresponding to this perspective. 2.2 “Public healthcare payer’s perspective” is a standard perspective that pertains to factors such as costs, comparator(s), and target populations within the range of the public healthcare insurance in Japan. 2.2.1 Even when an analysis is conducted from a perspective other than the “public healthcare payer’s perspective,” an analysis from the “public healthcare payer’s perspective” should also be submitted.” |
| Malaysia  2012 | Payer perspective with a supplementary societal or other perspective | “Pharmacoeconomic Guideline for Malaysia”  Ministry of Health Malaysia. Pharmaceutical Services Divisions  <https://www.pharmacy.gov.my/v2/sites/default/files/document-upload/pharmacoeconomic-guideline-malaysia.pdf> | “The study should be conducted from the perspective of the provider or funder in the applied setting. Patient and societal perspectives are encouraged. The perspective should be consistent for both cost and outcome components.” |
| MERCOSUR: Argentina, Brazil, Paraguay, Uruguay  2015 | Payer perspective with a supplementary societal or other perspective | “Guía Para Estudios de Evaluación Económica de Tecnologías Sanitarias”  Ministerio de Justicia y Derechos Humanos  <http://www.saij.gob.ar/25-internacional-guia-para-estudios-evaluacion-economica-tecnologias-sanitarias-rmr2015000025-2015-07-15/123456789-0abc-de5-2000-05102rserced> | “8. PERSPECTIVA DEL ANÁLISIS 8.1 Debe definirse la perspectiva del análisis de forma explícita, definiendo si es la perspectiva del sistema público de salud, de la sociedad, del sistema privado de salud, de un servicio sanitario (hospital, etc.) o del usuario / paciente. 8.2 Para decisiones involucradas en políticas públicas deberá preferirse la perspectiva del sistema público de salud. 8.3. En caso de seleccionar la perspectiva de la sociedad, se deben incluir todos los costos directos de la producción del servicio/ procedimiento y de los tiempos perdidos por los pacientes y sus familiares, además de los costos relacionados a la pérdida de productividad y muerte prematura.”  Translation:  *“8. PERSPECTIVE OF THE ANALYSIS 8.1 The perspective of the analysis must be explicitly defined, indicating if it is the perspective of the public health system, society, the private health system, a health service (hospital, etc.) or the user/patient. 8.2 For decisions involved in public policies, the perspective of the public health system should be preferred. 8.3. In case of selecting the perspective of the society, all the direct costs of the production of the service/procedure and of the time lost by the patients and their relatives, in addition to the costs related to the loss of productivity and premature death must be included.”* |
| Mexico  2015 | Payer perspective with a supplementary societal or other perspective | “Guía para la Conducción de Estudios de Evaluación Económica para la Actualización del Cuadro Básico y Catálogo de Insumos del Sector Salud en México.”  Comisión Interinstitucional del Cuadro Básico de Insumos del Sector Salud  <https://tools.ispor.org/PEguidelines/source/Mexico_GCEEE_2015.pdf> | “SI el objetivo del EEE es influir sobre el financiamiento público de insumos para la salud, como es el caso de la intención de incluir algún insumo al CBCISS, la perspectiva adoptada en el estudio debe ser la del sector público de salud como pagador, que está conformado por el conjunto de instituciones: SSA, IMSS, ISSSTE, CNPSS, SEDENA, SEMAR, PEMEX, SEDESA y DIF. Si se considera de relevancia pueden presentarse por separado el análisis que incluya resultados desde alguna perspectiva en particular (alguna institución en particular o la perspectiva social).”  Translation:  *“If the objective of the EEA is to influence the public financing of health supplies, as is the case with the intention of including some input to the CBCISS, the perspective adopted in the study should be that of the public health sector as a payer, which is formed by the set of institutions: SSA, IMSS, ISSSTE, CNPSS, SEDENA, SEMAR, PEMEX, SEDESA and DIF. If considered relevant, the analysis that includes results from any particular perspective (any particular institution or social perspective) can be presented separately.”* |
| Netherland  2016 | Societal perspective with a supplementary payer or other perspective | “Guideline for economic evaluations in healthcare”  National Health Care Institute  <https://english.zorginstituutnederland.nl/publications/reports/2016/06/16/guideline-for-economic-evaluations-in-healthcare> | “When conducting an economic evaluation, it is imperative to follow the reference case. This means that the economic evaluation is carried out and reported from the societal perspective. All relevant societal costs and benefits, irrespective of who bears the costs or to who the benefits go, should therefore be taken into account in the evaluation and reporting. In addition to the societal perspective the results can be presented from other perspectives (such as the healthcare perspective). The relevance of such a choice should be clearly underpinned.” |
| New Zealand  2015 | Payer perspective only | “Prescription for Pharmacoeconomic Analysis. Methods for cost-utility analysis”  Pharmaceutical Management Agency (PHARMAC)  <https://www.pharmac.govt.nz/assets/pfpa-2-2.pdf> | “3.3.1 Perspective: PHARMAC base-case cost-utility analyses are undertaken from the perspective of the funder. The key reason is that PHARMAC’s statutory objective requires it to achieve “the best health outcomes” “for eligible people”, and to do so “within the funding provided”.” |
| Norway  2018 | Societal perspective only  *(Stated as “Extended health care sector perspective”)* | “Guidelines for the Submission of Documentation for Single Technology Assessment (STA) of Pharmaceuticals”  Norwegian Medicines Agency (Legemiddelverket)  <https://legemiddelverket.no/Documents/English/Public%20funding%20and%20pricing/Documentation%20for%20STA/Guidelines_april_2018.pdf> | “In practice the guidance implies a form of *extended* health-service perspective. The following costs must be included (if relevant): (1) Treatment or prevention costs, paid by the health service or by the patient/relatives; (2) Transport costs linked to travelling to and from treatment, whether paid by the health service, or by the patient/relative; (3) Patient’s and relative’s use of time in connection with treatment. In accordance with the Priority-setting White Paper the following must not be included: (1) Productivity changes as a result of the intervention; (2) Consequences for patients’ future use of public services and receipt of public benefits/pensions; (3) Unrelated health service costs and savings. For example, the health service costs related to future unrelated illness will not be taken into consideration; (4) Tax expenses for public financing; (5) Public benefits, pension payments, value added tax and other transfer payments.” |
| Poland  2016 | Health care sector perspective with a supplementary societal or other perspective | “Wytyczne oceny technologii medycznych (HTA, ang. health technology assessment)”  Agencja Oceny Technologii Medycznych i Taryfikacji  <http://www.aotm.gov.pl/www/wp-content/uploads/wytyczne_hta/2016/20160913_Wytyczne_AOTMiT.pdf> | “Analizę należy wykonać z perspektywy podmiotu zobowiązanego do finansowania świadczeń ze środków publicznych (perspektywa płatnika publicznego) oraz złącznej perspektywy podmiotu zobowiązanego do finansowania świadczeń ze środków publicznych i świadczeniobiorców przy uwzględnieniu współpłacenia za technologie medyczne (łączna perspektywa płatnika publicznego i świadczeniobiorców). Jeżeli nie dochodzi do współpłacenia ze strony świadczeniobiorców lub jest ono znikome w zestawieniu z kosztem ponoszonym przez płatnika publicznego, można uwzględnić jedynie perspektywę płatnika publicznego. Powyższe perspektywy nie wykluczają przeprowadzenia w uzasadnionych sytuacjach dodatkowych analiz z innych perspektyw, np.: społecznej (przy uwzględnieniu kosztów pośrednich), świadczeniodawcy, finansów publicznych (przy uwzględnieniu świadczeń transferowych, takich jak renty, zasiłki).”  Translation:  *“The analysis should be performed from the perspective of the entity obliged to finance benefits from public funds (public payer perspective) and the joint perspective of the entity obliged to finance benefits from public funds and beneficiaries, taking into account co-payment for medical technologies (joint perspective of the public payer and beneficiaries). If there is no co-payment on the part of the beneficiaries or it is insignificant in comparison with the cost incurred by the public payer, only the perspective of the public payer can be taken into account. The above perspectives do not preclude conducting additional analyses in justified situations from other perspectives, e.g., social (taking into account indirect costs), the service provider, public finances (taking into account transfer benefits, such as pensions, benefits).”* |
| Portugal  1998 | Societal perspective only | “Guidelines for Economic Drug Evaluation Studies”  Ministry of Health and the National Health Service  <https://www.infarmed.pt/documents/281/1432055/PCAEC04_vering.pdf> | “The perspective should be that of society. This means considering the costs and consequences for the patient, for his or her family and also for third parties, i.e. public and private payers in particular. Society’s perspective should be broken down into other relevant points of view, with special attention to the third payers if they are the users of the study.” |
| Scotland  2017 | Payer perspective only | “Advising on New Medicines for Scotland”  Scottish Medicines Consortium (SMC)  <https://www.scottishmedicines.org.uk/media/2776/working-with-smc.pdf> | “The perspective adopted on costs should be that of the NHS in Scotland and social work. (referred to as Personal Social Services (PSS) in England).” |
| Singapore  2019 | Health care sector perspective only | “Drug Evaluation Methods and Process Guide”  Ministry of Health. Agency for Care Effectiveness (ACE)  [http://www.ace-hta.gov.sg/public-data/our-process-and-methods/ACE methods and process guide for drug evaluation (20 Dec 2019).pdf](http://www.ace-hta.gov.sg/public-data/our-process-and-methods/ACE%20methods%20and%20process%20guide%20for%20drug%20evaluation%20(20%20Dec%202019).pdf) | “The reference case analysis should only include direct healthcare costs from the perspective of the healthcare system. This includes payments out of the government’s and insurance providers’ healthcare budget as well as patients’ co-payments. Only patient-relevant, clinically meaningful outcomes should be included.” |
| Slovak Republic  2012 | Payer perspective with a supplementary societal or other perspective | “Metodická pomôcka pre vykonávanie farmako-ekonomického rozboru lieku, medicínsko-ekonomického rozboru zdravotníckej pomôcky a medicínsko-ekonomického rozboru dietetickej potraviny”  Ministerstvo Zdravotníctva Slovenskej Republiky  <https://www.health.gov.sk/?Dokumenty-Farmako-ekonomicky-a-medicinsko-ekonomicky-rozbor> | “7.6. Ak sa rozbor vykonáva z pohľadu nákupcu zdravotnej starostlivosti, k údajom o využívaní zdravotných zdrojov sa viaže najnižšia možná cena. Podobne aj finančná spoluúčasť pacientov sa vypočíta ako potrebná minimálna finančná spoluúčasť, ktorú musí pacient zaplatiť, aby dostal príslušný typ lieku, inej technológie alebo zdravotnej starostlivosti. Nákupca zdravotnej starostlivosti sa snaží maximalizovať prínos pre zdravie pri limitovaných zdrojoch a preto má záujem nahradiť aktuálnu nákladovo najefektívnejšiu alternatívu novou technológiou. Zároveň sa nákupcovia zdravotnej starostlivosti snažia ovplyvniť správanie poskytovateľov, aby tak uľahčili dodržiavanie nákladovo efektívnych postupov. 7.7. Zo spoločenského pohľadu sa môžu brať do úvahy náklady na produktivitu. Náklady na produktivitu sa vo východiskovej/základnej (base-case) analýze vypočítajú ako hrubá priemerná mzda pacientov v aktívnom veku. Takisto sa môže overiť vplyv použitia iných metód výpočtu. Náklady na produktivitu spadajú mimo zdravotnú starostlivosť a súvisia s vplyvom výkonu zdravotnej starostlivosti na produktivitu jednotlivcov. Pri použití spoločenskej perspektívy sa náklady na produktivitu použijú iba vtedy, ak sú signifikantné. V dôsledku neurčitosti metodológie výpočtu nákladov na produktivitu by sa mal vplyv použitia rôznych prístupov overiť v analýze senzitivity. Ak sú náklady na produktivitu vysoké, mali by sa uvádzať samostatne. Ide o doplnkovú analýzu, ktorú predkladateľ môže doplniť ku farmako-ekonomickému rozboru, avšak výsledky sú uvedené samostatne. […]Odporúča sa tiež oddeliť náklady hradené z verejných a zo súkromných zdrojov. Všeobecným princípom je, aby bola analýza prispôsobená pohľadu cieľových skupín, ktoré chcú osloviť autori rozboru. Pre krytie výdavkov z verejných zdrojov je to pohľad zdravotných poisťovní.”  Translation:  *“7.6. If the analysis is carried out from the perspective of a healthcare buyer, the lowest possible price is linked to data on the use of health resources. Similarly, the patient's financial participation is calculated as the minimum financial participation needed to be paid by the patient to receive the appropriate type of medicine, other technology or healthcare. The healthcare buyer seeks to maximize the health benefits of limited resources and is therefore keen to replace the current most cost-effective alternative with new technology. At the same time, healthcare buyers try to influence the behavior of providers to facilitate compliance with cost-effective practices. 7.7 From a social point of view, productivity costs can be taken into account. […] This is a complementary analysis that the applicant may supplement to the pharmaco-economic analysis, but the results are presented separately.* *It is also recommended to separate costs paid from public and private sources. The general principle is that the analysis should be adapted from the view of the target groups that the authors of the analysis want to address. It is the view of health insurance companies to cover expenditure from public sources.”* |
| Slovenia  2013 | Payer perspective with a supplementary societal or other perspective | „Pravilnik o razvrščanju zdravil na listo”  Zavod za zdravstveno zavarovanje Slovenije  [https://www.uradni-list.si/glasilo-uradni-list-rs/vsebina/112932/ - 6. člen](https://www.uradni-list.si/glasilo-uradni-list-rs/vsebina/112932/#6.%C2%A0člen) | “(1) Farmakoekonomska analiza se izvaja z vidika zdravstvenega zavarovanja. Analiza se lahko izvede tudi z družbenega vidika, zlasti kadar se lahko pričakuje pomembna razlika med družbenim vidikom in vidikom zdravstvenega zavarovanja. V tem primeru morata biti oba vidika prikazana ločeno.”  Translation:  *“(1) Pharmacoeconomic analysis is carried out from the point of view of health insurance. The analysis can also be carried out from a societal perspective, especially when a significant difference between the social aspect and the health insurance perspective can be expected. In this case, both aspects must be shown separately.”* |
| South Africa  2013 | Payer perspective with a supplementary societal or other perspective | “Guidelines for Pharmacoeconomic  Submissions”  Department of Health  <https://www.google.com/url?sa=t&rct=j&q=&esrc=s&source=web&cd=8&ved=2ahUKEwj5vabB1MLnAhXUYDUKHWlKAmAQFjAHegQICBAB&url=http%3A%2F%2Fwww.gpwonline.co.za%2FGazettes%2FGazettes%2F36118_1-2_Health.pdf&usg=AOvVaw1Fn_6AmTh-uQgSjRZQ6hfG> | Ordinarily, the Pricing Committee will only accept pharmacoeconomic submissions that adopt a third-party payer (i.e. a funder) perspective. Where a strong case can be made for adopting a broader perspective, the applicant must provide supporting argument, which at a minimum addresses the following: (a) justification for use of broader perspective, (b) rationale for additional costs to be included, (c) source of information to support additional costs, and (d) impact of this perspective on the results of the analysis.” |
| South Korea  2013 | Societal perspective only | “Korean Guidelines for Pharmacoeconomic Evaluation (Second and Updated Version)”  Health Insurance Review and Assessment Service (HIRA)  [https://link.springer.com/article/10.1007%2Fs40273-012-0021-6 - Tab2](https://link.springer.com/article/10.1007%2Fs40273-012-0021-6#Tab2) | “The first version recommended that an analysis should be conducted from a ‘societal’ perspective, meaning that all costs and effects should be considered [1, 10]. […] The advisory committee recommended that the revised guidelines should maintain the societal perspective but the terminology of the societal perspective be modified, rather than adopting the healthcare system perspective.  In the second version of HIRA’s guidelines, the societal perspective has been modified to be a ‘limited societal’ perspective to reduce unnecessary confusion and to be compatible with current practice patterns [8]. The terminology has been changed, but the content of the recommendation is consistent with that of the first version (Table 2).” |
| Spain  2010 | Both societal and payer perspectives | “Spanish Recommendations on Economic Evaluation of Health Technologies”  Spanish Ministry of Health and Social Policy  <https://link.springer.com/article/10.1007%2Fs10198-010-0244-4> | “The societal perspective considers all the outcomes that are significant from a societal perspective, and this is preferred as it represents the most general perspective. […] On the other hand, the perspective of the payer refers to all therapeutic benefits derived from the application of an intervention (compared with other alternatives) and the related costs incurred by the NHS when implementing or making new technology available. […] Although the societal perspective is recommended, the perspective of the payer (mainly the NHS) should also be considered, as it represents the main audience for an EEHT.” |
| Sweden  2017  ? | Societal perspective only | “Introduktion till hälsoekonomisk utvärdering”  Tandvårds- och läkemedelsförmånsverket (TLV)  <https://www.tlv.se/download/18.467926b615d084471ac3230c/1510316374332/TLVAR_2017_1.pdf>  <https://tlv.se/download/18.467926b615d084471ac3396b/1510316350460/introduktion-halsoekonomi.pdf> | “Den hälsoekonomiska analysen bör göras utifrån ett samhällsekonomiskt perspektiv. Detta innebär bland annat att alla relevanta kostnader och intäkter för behandling och sjuklighet oavsett vem de faller på (landsting, kommun, stat, patient, anhörig) bör beaktas. Ingående uppgifter ska beskriva svenska förhållanden.”  Translation:  *“The health economic analysis should be done from a socio-economic perspective. This means, among other things, that all relevant costs and revenues for treatment and morbidity regardless of who they fall on (county council, municipality, state, patient, relatives) should be taken into account. Incoming information should describe Swedish conditions.”* |
| Taiwan  2009 | Societal perspective with a supplementary payer or other perspective | “Guidelines of Methodological Standards for Pharmacoeconomic Evaluations in Taiwan”  Taiwan Society for Pharmacoeconomics and Outcomes Research  <https://tools.ispor.org/PEguidelines/source/2006_PEG_EN_2009.pdf> | “Pharmacoeconomic evaluations are recommended to be conducted and reported from the societal perspective; then analyses for different target audiences are presented separately.” |
| Thailand  2017 | Societal perspective only | “Guidelines for health technology assessment in Thailand (second edition): Recommendations for defining the scope of HTA”  Department of Health, Ministry of Public Health  <http://www.hitap.net/wp-content/uploads/2017/06/Thai-HTA-guideline-UPDATES-Jmed-with-Cover.pdf> | “The societal perspective should be adopted.” |
